# Radon groundwater in a radon-prone area: possible uses and problems: an example from SW part of Kłodzko Valley, Sudetes, SW Poland

**DOI:** 10.1007/s10653-022-01212-0

**Published:** 2022-02-08

**Authors:** Tadeusz A. Przylibski, Stanisław Staśko, Elżbieta Domin

**Affiliations:** 1 Faculty of Geoengineering, Mining and Geology, Division of Geology and Mineral Waters, Wrocław University of Science and Technology, Wybrzeże S. Wyspiańskiego 27, 50-370 Wrocław, Poland; 2 Faculty of Earth Sciences and Environmental Management, Institute of Geological Sciences, Department of General Hydrogeology, University of Wrocław, Pl. Maksa Borna 9, 50-205 Wrocław, Poland

**Keywords:** Radon, Radon water, Medicinal water, Drinking water, Radon potential, Radon-prone area

## Abstract

The paper describes research aimed at expanding scientific knowledge of radioactive isotope ^222^Rn occurrence in groundwaters flowing in crystalline rocks, including its spatial and temporal changes. The research, conducted in an area characterized by medium radon potential, was intended to determine the values of ^222^Rn activity concentration in groundwater in this type of areas. The ^222^Rn activity concentration in groundwaters discharged from investigated springs oscillated between 35.3 and 272.0 Bq/L. The authors discovered possible prevalence of radon groundwaters in areas with medium radon potential, which is the reason why all groundwaters intended for human consumption or household use in such areas should be subject to obligatory monitoring of ^222^Rn activity concentration. In the event of identifying occurrence of waters with ^222^Rn activity concentration of at least 100 Bq/L, their purification by removing radon is necessary before they are supplied to a water distribution network. At the same time, the research area can be regarded as an area with potentially medicinal radon water occurrence. Therefore, in areas with medium radon potential, groundwaters which are not suitable as a source of drinking water due to very high ^222^Rn activity concentration in them can be used as medicinal radon waters in therapeutic treatments.

## Introduction

One of the most important radioactive isotopes giving groundwaters their radioactive properties is the most durable radon isotope, ^222^Rn (Chau et al., 2011) with the half-life of 3.82146 days (Bellotti et al., 2015). ^222^Rn activity concentration in groundwater is very changeable, with differences reaching nine orders of magnitude (Girault et al., 2018). In areas with complicated geological structure, waters from intakes lying as close as about a dozen metres apart from one another may exhibit ^222^Rn activity concentration differences reaching even 2–4 orders of magnitude (Cho & Choo, 2019; Przylibski, 2005; Przylibski et al., 2008; Sukanya et al., 2021). This is due to the fact that the area from which a well or a spring is recharged with ^222^Rn lies at a distance from several to a few dozen metres from the intake or groundwater outflow. Only sporadically (e.g. in karst reservoirs), ^222^Rn can reach a water intake from a distance larger than 100 m. This is directly related to the velocity of water flow in an aquifer during the lifetime of ^222^Rn nuclide, i.e. about 38.2 days (Przylibski, 2000b, 2005). In areas with no occurrence of uranium deposits or large mineralization zones, the highest values of ^222^Rn activity concentration are recorded in waters from shallow and transitional flow systems. This is due to the fact that with uniform concentration of parent ^226^Ra in the reservoir rock, this is the emanation coefficient which plays the dominant role in supplying ^222^Rn to groundwater. As it is largely dependent of rock fracturing, its values are usually the highest near the earth’s surface. Consequently, the largest amounts of ^222^Rn are dissolved at small depths, usually in groundwater drainage zones (Freiler et al., 2016; Martins et al., 2020; Przylibski, 2000a, 2005, 2011; Thivya et al., 2017).

In radon-prone areas or areas with high or medium radon potential, groundwaters with increased radon content are common (Pinti et al., 2014; Przylibski et al., 2007). In Poland, such areas occur chiefly in Lower Silesia, particularly in the Sudetes, i.e. the SW part of the country (Przylibski, 2015). In these areas, waters with increased ^222^Rn content are frequently the only source of water for households, including drinking water. They are also used in public water intake points supplying municipal systems providing towns and villages with water intended for human consumption. On the other hand, such waters can be used in health resorts as medicinal radon waters (Przylibski, 2005, 2018; Przylibski et al., 2007; Seminsky & Seminsky, 2016).

In Poland, like in the other European Union countries, the maximum allowable ^222^Rn activity concentration in water intended for human consumption is set by law at the level of 100 Bq/L (Council Directive, 2013; Ordinance of the Minister of Health, 2017). At the same time, waters with ^222^Rn activity concentration over 74 Bq/L can be, in accordance with Polish law, recognized as medicinal (Act, 2011) and they are often treated as potentially medicinal (Przylibski, 2018; Przylibski et al., 2007, 2008). Such dual approach to groundwaters enriched in radioactive ^222^Rn isotope occurring particularly in radon-prone areas or areas with high or medium radon potential is due to adopting different theories of the effect of ionizing radiation on the human body. Adopting either the radiation hormesis theory or the linear hypothesis leads to either using radon waters and the ^222^Rn dissolved in them for balneotherapeutic and radon-therapeutic treatments or to the necessity of removing radon from water before it is used in households (Przylibski, 2005, 2018). Not only in Poland, but also in many countries, especially in Central Europe, radon waters are used for balneotherapeutic treatments in a variety of health and spa resorts. The largest number of places where radon waters, or radon present in the atmosphere of caves or mines, are used for medicinal purposes are found in Germany, Austria, Hungary, Czech Republic, Italy, France, Greece, Bulgaria, Bosnia and Herzegovina, Romania, and Russia, and outside Europe also in the USA, China, Japan and Chile (Becker, 2004; Cucu et al., 2017; Erickson, 2006, 2007; Falkenbach et al., 2005; Franke et al., 2000, 2007; Kapetanović et al., 2013; Moder et al., 2011; Nagy et al., 2008; Persianova-Dubrova et al., 2012; Piao et al., 2020; Przylibski, 2018; Somlai et al., 2007; Vogiannis et al., 2004; Voronov, 2004; Zdrojewicz & Strzelczyk, 2006). However, it is not the ongoing scientific dispute on the application of radon waters in balneotherapeutic treatments in light of radiation hormesis theory which is the subject of this study. This aspect of radon occurrence in the environment, including groundwaters, has been repeatedly discussed more broadly, e.g. by Becker (2004) and Przylibski (2018), as well as in the publications cited in these two review papers.

**Fig. 1:**
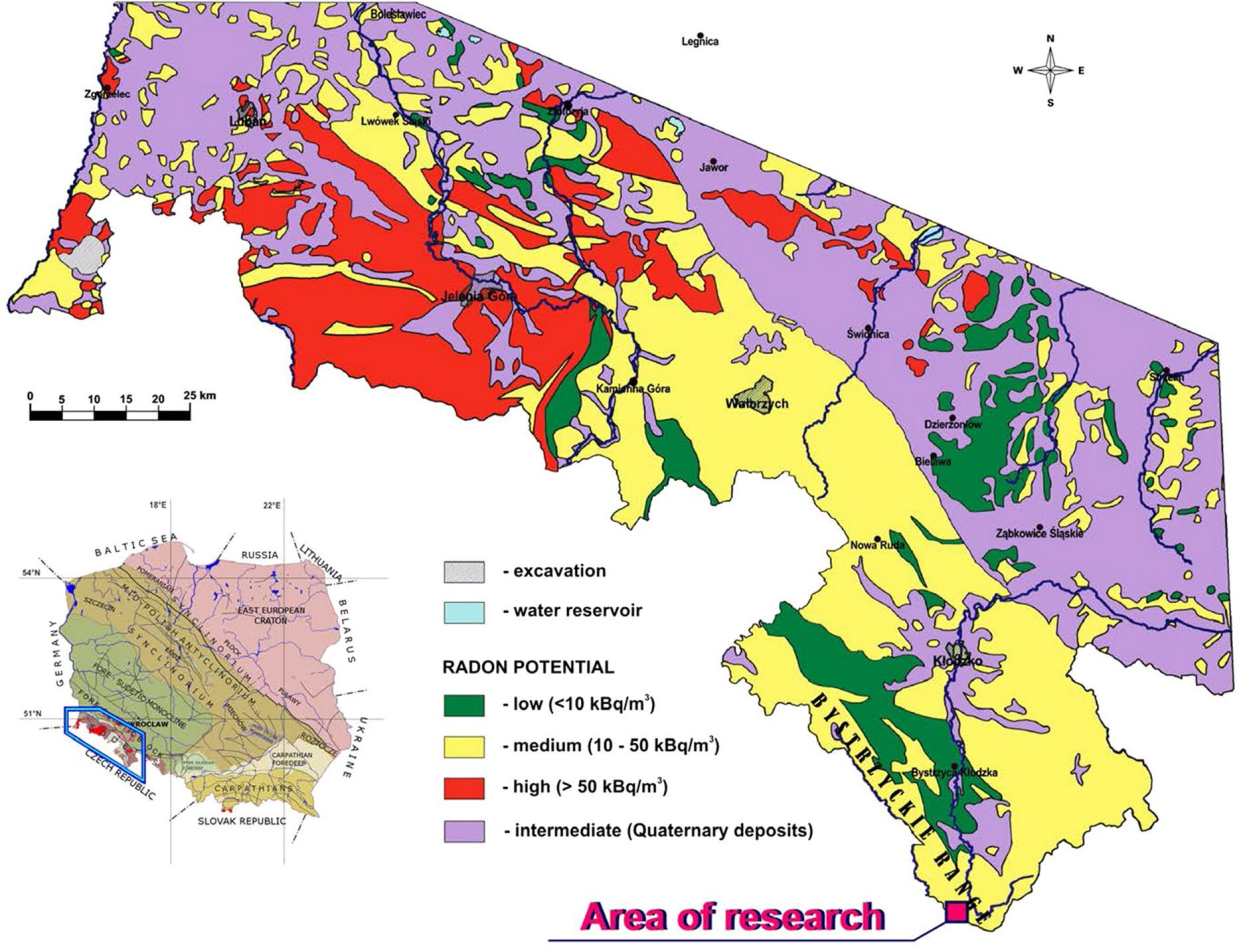
Research area on a map of radon potential of the Sudetes and selected geological units of the Fore-Sudetic block (according to Wołkowicz, 2007)

**Fig. 2:**
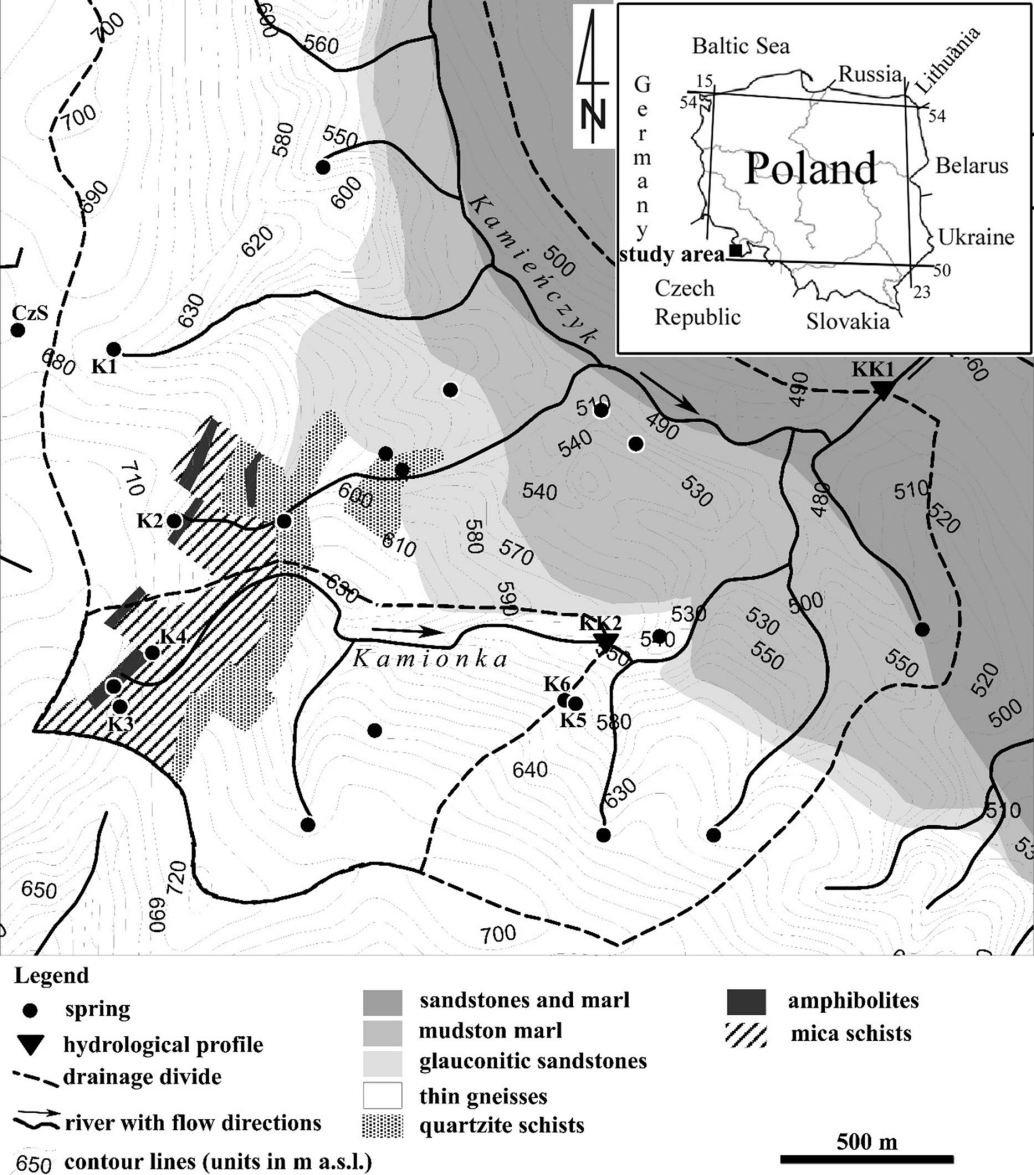
Study area illustrating the network of observation and sampling points with geological structure background according to Sawicki (1995), and Buczyński and Staśko (2016)

The authors’ research concerned with ^222^Rn behaviour in fresh groundwater environment was conducted in the SW part of the Kłodzko basin, a part of the Sudetes, the mountain system stretching in the SW part of Poland along the border with the Czech Republic (Figs. 1 and 2). The aim of this research was to recognize in detail the occurrence of potentially medicinal radon waters in crystalline rocks in the southern part of the Bystrzyckie Mountain range (Fig. 1). An added value of this research was to recognize spatial and temporal activity concentration changes of ^222^Rn in local and intermediate groundwater flow system in hard rocks of the Kłodzko basin (Ciężkowski & Przylibski, 1997; Przylibski & Żebrowski, 1999; Przylibski, 2000c, 2015; Przylibski et al., 2002a, 2002b, 2007, 2014; Walencik-Łata et al., 2016). On the other hand, the research was planned in a way making it possible to assess spatial changes in ^222^Rn activity concentration in shallow groundwaters within one type of crystalline reservoir rocks, i.e. gneisses and mica schists of the Orlica-Bystrzyca metamorphic complex. Finally, the research was conducted in an area with medium radon potential in order to determine the value of ^222^Rn activity concentration in groundwaters in this type of areas in view of a possibility of using groundwaters from such areas as drinking water for their populations.

The authors’ principal aim is to demonstrate, based on an example of a small area built of various crystalline rocks, that shallow circulation groundwaters occurring in such reservoir rocks may contain dissolved ^222^Rn in a wide range of concentrations. Therefore, these waters might require de-radoning before being used as waters intended for human consumption. At the same time, such waters may be used as a potential raw material for balneotherapy treatments in spa resorts. The results of our research, although conducted in a small area of the Sudetes, will be certainly representative of other areas on all continents with geological structure dominated by crystalline (metamorphic and igneous) rocks. This especially refers to areas with a similar—high and medium radon potential. It is related to the geochemical characteristics of rocks such as granite, gneiss (especially orthogneiss), or various types of crystalline schists. If other (sedimentary) rocks contain increased concentrations of uranium and radium, also such areas will be classified as areas with medium or high radon potential. For this reason, areas with medium or high radon potential will be very similar in terms of the presence of groundwater with high and diversified radon content.

## Geological and hydrogeological setting

The study area is located in SW Poland, in the Bystrzyckie Mts, a part of the Sudetic mountain system, and belongs to the geological unit of the Orlica-Śnieżnik dome. Different types of gneisses and mica and quartzite schists associated locally with amphibolite of the Proterozoic—Upper Cambrian age, 514–490 Ma (Żelaźniewicz, 2015), build up the catchment area (Don et al., 1990; Mazur et al., 2006, 2010; Sawicki, 1962, 1995; Szczepański, 2010). The gneisses come in different textures: laminar, auger and flaser (Fig. 2).

Groundwaters flow out chiefly in springs associated with tectonic zones. They occur in fractured brittle base rocks and in cover, as observed by Krasny (1993) and Lassachagne et al. (2001).

The upper Kamieńczyk valley is located above numerous tectonic zones consisting of faults running perpendicular or along the valley axis. Reddish-brown eluvial deposits, sometimes over two metres thick, cover the levelled-out areas and slopes near mountain peaks. The main part of the study area, located at the altitude of 500–710 m a.s.l., belongs to the Nysa Kłodzka river watershed, while the spring Czerwony Strumień is located on the other side of the local water divide and belongs to the Dzika Orlica watershed (Fig. 2).

Groundwater occurrence in crystalline hard rocks of the Sudetes is characterized by three water-bearing zones differing in storage and transmissivity properties (Staśko, 2002). The upper zone, or the weathering cover, is characterized by highly varied thickness (from 1 to 10 m), relatively high specific yield *μ* = 0.18 and low hydraulic conductivity *k* = 0.1 m/d. The middle zone is made up of densely fractured rocks with thickness ranging from 10 to 50 m. It is characterized by lower capacity, within the range of 0.008–0.05, and higher hydraulic conductivity, around 1 m/d. The deepest zone, with the lowest capacity (*μ* = 0.0001–0.001) and conductivity (*k* = 0.001–0.1 m/d) consists of deep faults and is associated with fault systems with depths ranging from 100 to 500 m. The crystalline hard rocks show regular fracture and fissure systems varying in extent, both vertically and horizontally. Due to differences in terrain morphology, local and transitional groundwater flow could be distinguished in such terrains and conditions (Buczyński & Staśko, 2016). However, a zone of intensive groundwater circulation is related to the most fractured bed with weathered cover. Detailed research and hydrogeological observations in mountainous terrain (Staśko & Tarka, 2002) have indicated the complexity of groundwater occurrence and complicated groundwater circulations. Fractured hard rocks are preferential groundwater flow zones, rather than typical aquifers. A large number of springs and direct outflows to river bed sediments are a typical feature of groundwater discharge. A limited number of wells is characteristic of such terrain. The springs under observation (Fig. 2) showed low discharge and relatively stable outflow and water temperature (Table 1).

**Table 1:** Characteristics of examined springs and outflowing groundwaters based on periodic measurements in years 2007–2016

Location	Intake/spring (symbol)	Reservoir rocks	Sampling date	Activity concentration ± measurement uncertainty of		Type of groundwater by dissolved ^222^Rn content*	Q L/s	pH –	EC μS/cm	T °C
				^222^Rn	^226^Ra					
				Bq/L						
Czerwony Strumień	Czerwony Strumień(CzS)	Thin gneisses/quartzite	08.02.2014	93.9 ± 1.2	0.07 ± 0.06	Low-radon water	1.20	5.02	58	8.1
			02.05.2014	95.4 ± 4.9	0.06 ± 0.06		1.12	5.18	55	7.3
			25.08.2014	80.3 ± 1.1	< LLD		0.72	5.04	54	7.8
			09.11.2014	98.3 ± 2.0	< LLD		1.20	5.3	56	9.2
			10.02.2016	92.5 ± 1.2	< LLD		1.60	5.42	59	8.7
			21.05.2016	94.8 ± 0.9	< LLD		1.14	5.63	52	7.3
			25.11.2016	52.6 ± 1.0	< LLD		0.31	5.2	54	9.7
Kamieńczyk	Międzylesie intake (K1)	Thin gneisses	16.08.2007	158 ± 8	N.a	Radon water	0.38	5.9	66	7.8
			25.11.2016	196 ± 3	< LLD		1.02	5.72	75	8.4
	Teresa’s spring (K2)	Thin gneisses/ mica schists	16.08.2007	35.3 ± 3.1	N.a	Low-radon water	0.18	7.14	56	8.2
			25.11.2016	39.3 ± 0.8	< LLD		0.41	6.14	83	7.9
	Graniczne spring (K3)	Mica schists/amphibolites	25.11.2016	192 ± 2	< LLD	Radon water	0.04	5.28	20	8.4
	Janusz’s spring (K4)	mica schists/amphibolites	16.08.2007	169 ± 8	N.a	Radon water	0.05	5.94	120	7.6
			13.01.2008	238 ± 7	N.a		0.21	6.8	78	6.1
			09.03.2008	272 ± 9	N.a		0.50	6.12	66	6.6
			07.07.2008	197 ± 6	N.a		0.05	6.6	46	6.8
			11.11.2008	167 ± 5	N.a		0.01	6.34	71	7.7
			08.02.2014	223 ± 2	< LLD		0.16	5.6	82	7.7
			02.05.2014	201 ± 3	0.07 ± 0.06		0.10	5.49	78	7.3
			25.08.2014	184 ± 1	< LLD		0.07	5.28	63	8.3
			10.11.2014	202 ± 4	< LLD		0.11	5.64	81	8.7
			10.02.2016	214 ± 2	< LLD		0.07	5.4	76	8.5
			21.05.2016	185 ± 2	< LLD		0.03	5.74	81	7.2
			25.11.2016	137 ± 2	< LLD		0.03	5.85	78	8.7
	Kamienne spring (K6)	Thin gneisses	16.08.2007	61.2 ± 4.4	N.a	Low-radon water	0.11	5.81	67	7,0
			25.08.2014	70.4 ± 1.3	< LLD		0.21	5.29	68	7.5
			10.11.2014	56.5 ± 1.0	< LLD		0.35	5.37	68	7.8
			11.02.2016	62.6 ± 1.0	< LLD		0.75	5.03	68	8.2
			21.05.2016	70.1 ± 0.7	< LLD		0.14	5.23	64	6.9
			25.11.2016	58.6 ± 1.5	< LLD		0.20	5.25	73	8.8

*—classification by Przylibski (2005). Waters considered to be potentially medicinal radon waters are underlined;

< LLD—values below lower limit of detection;

N.a.—not analysed

An analysis of the chemical composition of groundwaters in the study area has revealed varying hydrodynamic conditions and chemical parameters typical of local groundwater flow systems (Modelska et al., 2015). Typical groundwater characteristics in the upper zone of the catchment are low pH value (5.8) and SO_4_–HCO_3_–Na–Ca or SO_4_–HCO_3_–Ca–Na water composition. In the lower part of the catchment, HCO_3_–SO_4_–Ca–Na or HCO_3_–SO_4_–Na–Ca water type prevails. In the lowest zone, higher pH value (6.9) and a lower value of Si content are observed. The groundwater shows very low total mineralization with TDS ranging from 36 to 84 mg/L and low acidity (5.75–7.12). The hydrogeochemical background of ^222^Rn in the area of the Orlica-Bystrzyca metamorphic complex constituting the western part of the Orlica-Śnieżnik Dome geological unit is 8–309 Bq/L (Adamczyk-Lorenc, 2007).

**Fig. 3:**
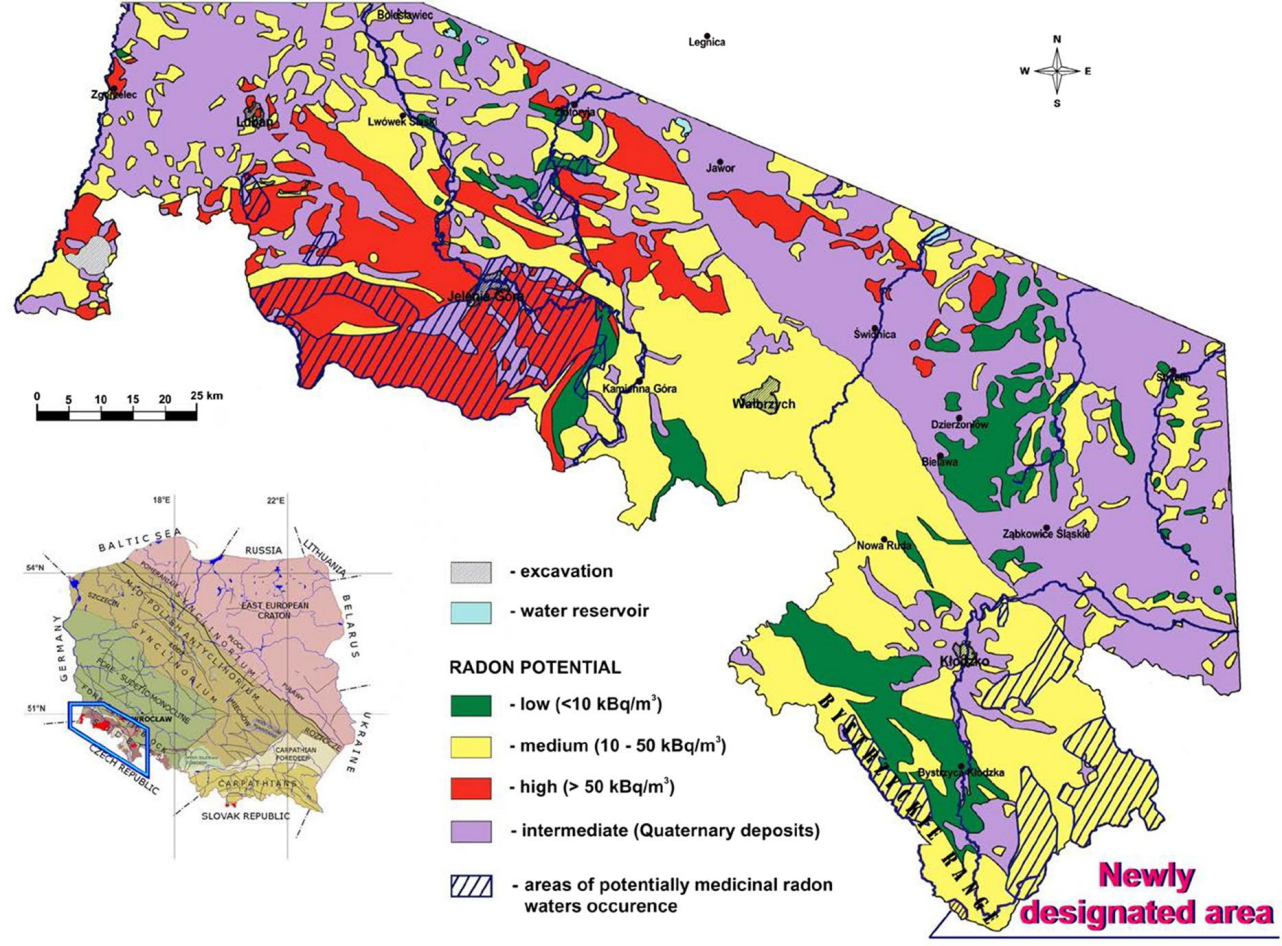
A new, second area with potentially medicinal radon water occurrence in the Orlica-Bystrzyca metamorphic complex against a map of radon potential of the Sudetes and selected units of the Fore-Sudetic block (according to Wołkowicz, 2007) and the remaining, previously identified areas with potentially medicinal radon water occurrence in the Sudetes (according to Przylibski et al., 2007)

The study area lies in the southern part of the Orlica-Bystrzyca metamorphic complex, where ^222^Rn activity concentration values recorded in groundwaters range from 0.3 to 448 Bq/L, with the arithmetic mean for measurements performed at 37 points equalling 105 Bq/L and the median—66.6 Bq/L (Przylibski et al., 2007). So far, potentially medicinal radon waters have been identified in one area in the Orlica-Bystrzyca metamorphic complex (Fig. 3) (Przylibski et al., 2007).

## Measurement and calculation methods

Spring discharge was evaluated using a volumetric method with an error of ± 5%. Groundwater temperature was measured directly in water outlets with a calibrated thermometer with the accuracy of 0.05 °C. Measurements of electrical conductivity and pH in water samples were taken in a laboratory by means of electronic equipment produced by Elemetron. The uncertainties of these measurements are ± 0.05 µS/cm and 0.05 pH unit, respectively.

Fieldwork included collecting water samples with the aim of determining the activity concentration of ^222^Rn in water. Every time, three 10 cm^3^ water samples were taken with a disposable syringe and transferred to 3 glass scintillation vials filled with 10 cm^3^ of liquid scintillator InstaFLUOR Plus. The vials were subsequently sealed and shaken vigorously to enable radon dissolved in water to penetrate into the layer of scintillator, which is immiscible with water and where radon is better dissolved. This ensured the registration of all radioactive decays by a spectrometer. The scintillation vials containing the samples were later transported to a laboratory, where they were placed in an ultra-low background liquid-scintillation spectrometer *α*/*β* Quantulus 1220, which was used to perform further measurements.

Spectrometer measurements of ^222^Rn activity concentration are based on the external photoelectric effect and on the scintillation phenomenon. Scintillation enables ionizing radiation detection by registering flashes of light generated at the moment of alpha particles, being the effect of radioactive transformations of ^222^Rn and its daughters, reaction with the scintillator. Flashes of light are registered as a measurable electric current impulse thanks to the photoelectric effect. The number of registered impulses is directly proportional to the number of radioactive transformations occurring in the sample. The spectrometer is calibrated with certified reference standards in the form of solutions containing ^226^Ra with strictly specified activity concentration. These solutions were prepared on the basis of a certified standard source of ^226^Ra with a concentration of 0.914 µg/g. The source was purchased from the producer, EUROSTANDARD.CZ spol. S r.o. from Prague. It is certified by the Czech Metrology Institute in Brno. The activity of this source on the day of purchase (May 26, 2014) was 1.67869 × 10^5^ Bq. Eighteen standard solutions were prepared by the dilution method, and the precision was controlled by weighing out the solutions with an analytical balance with the accuracy of mass determination up to 0.0001 g. The standard solutions covered the activity concentration range of ^222^Rn from 0.0044 to 5036 Bq/L. The number of counts (impulses) recorded by the spectrometer is converted into the activity concentration of ^222^Rn in the water sample on the basis of the equation of the calibration curve obtained from measurements of standard solutions. Using the radioactive decay equation, this result is converted into the time of sampling the water in the field.

Each sample was subject to nine one-hour long measurements, which produced the total of 27 ^222^Rn activity concentration results. Thanks to this, statistical treatment of result enabled reducing determination uncertainty to less than 1%. The lower detection limit of the applied method is 0.05 Bq/L (about twice the number of counts recorded by the spectrometer as compared to the number of background counts). The same samples as for ^222^Rn analyses are used to measure ^226^Ra activity concentration and are subjected to the same tests. However, the measurement begins only after the time required to achieve a state of radioactive equilibrium between ^222^Rn and ^226^Ra in the sample.

Prior to determining the hydrogeochemical background of ^222^Rn, preliminary data analysis was conducted. It was aimed at verifying and rejecting from the population of data the ^222^Rn activity concentration values containing a gross error as well as extreme values and outliers.

In view of the population size, data analysis in terms of the presence of gross errors was performed with Graf’s statistical test (Szczepańska & Kmiecik, 1998). It demonstrated the gross error to be the value *x*_*d*_ lying outside *x*_*excl*_ ± *4σ*_*excl*_ interval, in which *x*_*excl*_ and *4σ*_*excl*_ are the arithmetic mean and the standard deviation, respectively, obtained after excluding the doubtful result *x*_*d*_ from the data set (Danzer & Lube, 1996). The extreme values are situated at the distance of three interquartile ranges (3 × H) from the lower and the upper quartiles, and the outliers—at the distance of 1.5 interquartile ranges (1,5 × H) (Janica, 2002). In the set of 43 analysed data, no gross errors or extreme values were recorded. One outlier, being the minimum value of the set (0.3 Bq/L), was identified and rejected in the course of further calculations aimed at determining the hydrogeochemical background of ^222^Rn.

After excluding the abovementioned values, the hydrogeochemical background of ^222^Rn was calculated. To this end, the authors used the most reliable computational method *Z* ± 1.28 × *σ* based on the arithmetic mean (*Z*) and standard deviation (*σ*) values (Adamczyk-Lorenc, 2007). As the data were characterized by log-normal distribution, all the operations were performed on logarithmized data.

## Results and discussion

Detailed results of measurements performed in groundwaters from six springs in 2007–2016 are collected in Table 1. According to Przylibski’s classification (2005, 2011), waters from springs CzS, K2 and K6 are low-radon waters, i.e. containing from 10 to 99.9(9) Bq/L of dissolved ^222^Rn. Waters flowing out in the other springs, i.e. K1, K3 and K4, are radon waters, i.e. containing from 100 to 999.9(9) Bq/L of dissolved ^222^Rn. It appears that in the area of about 4 km^2^, groundwaters flowing out in springs lying from several hundred metres to almost 2 km apart (cf. Fig. 2) differ in the activity concentration of ^222^Rn dissolved in them by an order of magnitude. This observation confirms the mosaic-like image of spatial distribution of the concentration of ^222^Rn dissolved in groundwaters in areas built of crystalline rocks. This fact has already been described in earlier publications, including Przylibski, 2005, 2011; Przylibski et al., 2008. In the studied area (cf. Fig. 2), low-radon waters flow out at the contact of thin gneisses with quartzite and from thin gneisses in springs CzS and K6, respectively, or from the contact zone between thin gneisses and mica schists in spring K2. Radon waters, with ^222^Rn concentration higher by an order of magnitude, also flow out in springs occurring within thin gneisses (spring K1) or at the contact between mica schists and amphibolites (springs K3 and K4). This means that ^222^Rn activity concentration in the studied groundwaters is probably determined chiefly by the presence of brittle tectonic deformations in the vicinity of springs, while the role of lithology is only minor.

The values of ^222^Rn activity concentration measured in groundwaters from all the studied springs fall within the range of 35.3–272.0 Bq/L. The ^222^Rn originates wholly from the dissolution in these waters of gas formed in their reservoir rocks as a result of parent ^226^Ra decay. The ^226^Ra^2+^ ion content in these groundwaters, which could be the source of ^222^Rn, is virtually negligible (cf. Table 1). The measured values of ^222^Rn activity concentration lie within the hydrogeochemical background of ^222^Rn determined for groundwaters of the Orlica-Bystrzyca metamorphic complex, i.e. 8–309 Bq/L (Adamczyk-Lorenc, 2007). The values measured hitherto in groundwaters of this geological unit fall within the range of 0.3–448 Bq/m^3^. For 37 measurement points, the mean value of ^222^Rn activity concentration in groundwaters of the Orlica-Bystrzyca metamorphic complex reached 105 Bq/L, and the median—66.6 Bq/L (Przylibski et al., 2007). The measurements taken by the authors in six springs make another 16% of groundwater sampling in the area of the Orlica-Bystrzyca metamorphic complex. This enables a recalculation of the hydrogeochemical background of ^222^Rn in groundwaters of this unit and providing the basic descriptive statistics of ^222^Rn activity concentration in groundwaters of the Orlica-Bystrzyca metamorphic complex. The new parameters characterizing the concentration of ^222^Rn dissolved in groundwaters of the Orlica-Bystrzyca metamorphic complex, based on archival data supplemented with the mean values obtained for groundwaters flowing out in the 6 springs studied by the authors, are shown in Table 2.

**Table 2:** Hydrogeochemical background of ^222^Rn in groundwaters in the Orlica-Bystrzyca metamorphic complex and selected descriptive statistics characterizing the activity concentration of ^222^Rn dissolved in groundwaters in this geological unit

Hydrogeochemical background of ^222^Rn	10 ÷ 317
Number of data	43
Mean	107.6
Minimum	0.3
Maximum	448.0
Range	447.7
Median	67.0
Standard deviation	119.9
Kurtosis	1.8
Skewness	1.6

The values of all statistical parameters expressed in Bq/L

Table 3 presents selected descriptive statistics characterizing ranges of ^222^Rn activity concentration changes in particular springs. The values shown in Table 3 indicate that differences between the mean and the median are hardly significant. This indicates the probably normal character of the distribution of ^222^Rn activity concentration values in springs CzS, K4 and K6. In the other springs, fewer than 6 data are available, making determination of data distribution impossible. At the same time, the presented data suggest that groundwaters flowing out in particular springs do not change their type in terms of ^222^Rn content. This means that these are always low-radon waters that flow out in springs CzS, K2 and K6, and radon waters—in the remaining springs (K1 and K4). The radon water spring K3 has been sampled only once, which makes it impossible to investigate temporal changes in ^222^Rn activity concentration in groundwater flowing out of it. The widest range of ^222^Rn activity concentration changes in time has been recorded in radon waters flowing out in spring K4. In low-radon water springs CzS and K6, these changes were smaller (cf. Tables 1 and 3).

**Table 3:** Selected descriptive statistics characterizing ^222^Rn activity concentration in groundwaters from the studied springs

Intake/spring (symbol)	Number of data	Mean	Minimum	Maximum	Range	Median	Standard deviation
		[Bq/L]					
Czerwony Strumień (CzS)	7	86.8	52.6	98.3	45.7	93.9	16.2
Międzylesie intake (K1)	2	177.0	158.0	196.0	38.0	177.0	–
Teresa's spring (K2)	2	37.3	35.3	39.3	4.0	37.3	–
Graniczne spring (K3)	1	192.0	–	–	–	–	–
Janusz's spring (K4)	12	199.1	137.0	272.0	135.0	199.0	35.5
Kamienne spring (K6)	6	63.2	56.5	70.4	13.9	61.9	5.8

**Fig. 4:**
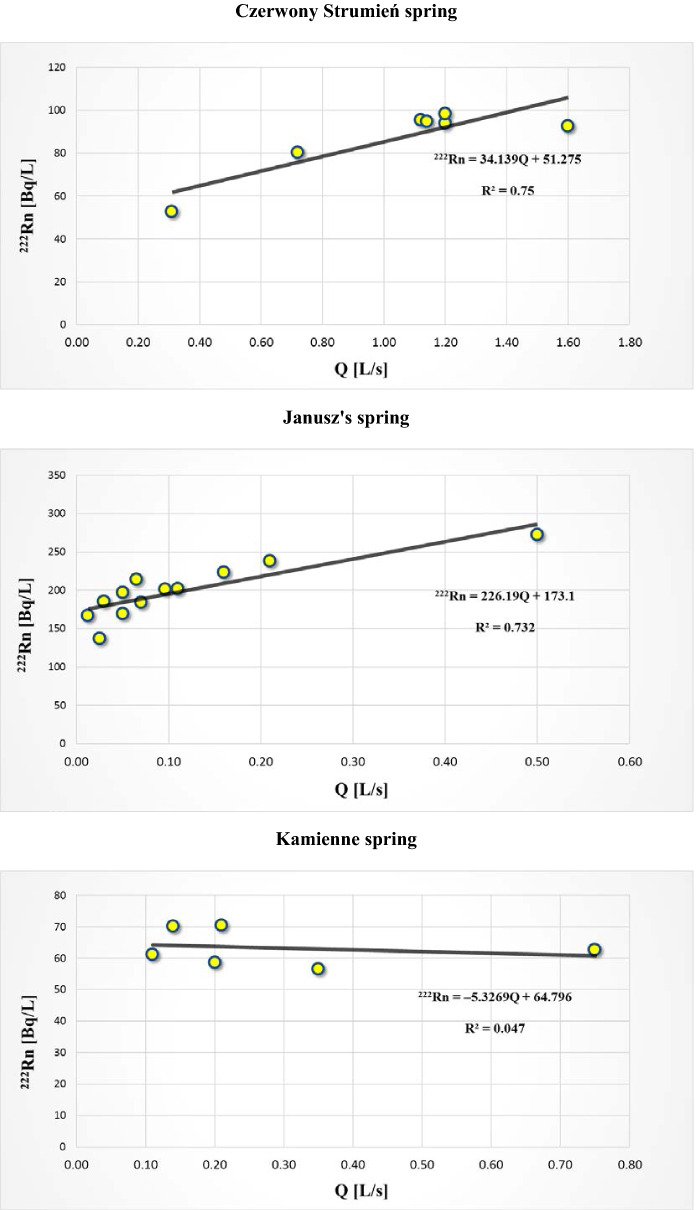
Correlation between the activity concentration of ^222^Rn dissolved in groundwaters flowing out in Czerwony Strumień (CzS), Janusz’s (K4) and Kamienne (K6) springs and their discharge Q

Table 4 contains values of linear correlation coefficient between ^222^Rn activity concentration and some of the other measured parameters: discharge Q, pH, electrolytic conductivity EC and water temperature T of low-radon waters flowing out in springs CzS and K6, and of radon water flowing out in spring K4. A statistically significant strong correlation between ^222^Rn activity concentration and spring discharge Q has been identified in low-radon water and radon water in springs CzS and K4, respectively. An increase in discharge is accompanied by a rise in ^222^Rn activity concentration (Fig. 4). This indicates a rise in radon content with an increase in water flow intensity in the water-bearing zone near an intake. A rise in water flow velocity results in the ability of ^222^Rn released from reservoir rocks and dissolved in flowing groundwater to travel with this water over a longer distance before its decay. This means that the dissolved ^222^Rn may reach a spring from a longer distance; hence, its activity concentration is higher, leading to an increase in the capacity of the recharge zone supplying the spring with ^222^Rn. This observation is consistent with previous findings from the Sudetes, including those from the areas of Świeradów-Zdrój and Lądek-Zdrój (Przylibski, 2000b, 2005). However, this regularity has not been confirmed by observations of spring K6 in the studied area. In this spring, no correlation has been found between ^222^Rn activity concentration and discharge Q (cf. Table 4 and Fig. 4). The recharge zone supplying this spring with groundwater and ^222^Rn requires further investigation. At the same time, in all the springs (CzS, K4 and K6), a statistically significant inversely proportional correlation was found between ^222^Rn activity concentration and water temperature T in the spring (cf. Table 4). Although it should be noted that annual changes in water temperature are small and are 2–3 °C, while the changes in air temperature are around 40 °C. This observed correlation means that the lower the temperature of water, the higher the activity concentration of the ^222^Rn dissolved in it. This observation is consistent with Henry’s law, stating that solubility of gases decreases with an increase in water temperature. However, when it comes to radon and groundwaters, water is practically never saturated with radon (e.g. Walia et al., 2003, vide Przylibski, 2005). Therefore, it is unlikely that such a simple mechanism, based on water temperature change, is responsible for variation in the amount of ^222^Rn dissolved in it. The causes of such a relationship should be sought in the groundwater flow system. It is likely that infiltration waters cause the expansion of reservoir rock washing zone and an increase in the velocity of water flowing to the spring, which results in an increase in ^222^Rn activity concentration in water appearing in the spring. However, the confirmation of such a mechanism would require a much bigger number of more accurate measurements as well as research on water residence time in the aquifer.

**Table 4:** Values of linear correlation coefficient between ^222^Rn activity concentration in groundwaters and spring discharge *Q*, pH of water, electrolytic conductivity EC of water and temperature *T* of water from the studied springs CzS, K4 and K6

Location	Intake/spring (symbol)	*Q*	pH	EC	*T*
Czerwony Strumień	Czerwony Strumień (CzS)	**0.87**	0.26	**0.31**	**− 0.55**
Kamieńczyk	Janusz’s spring (K4)	**0.86**	0.18	− 0.24	**− 0.57**
Kamieńczyk	Kamienne spring (K6)	− 0.22	− 0.22	**− 0.58**	**− 0.55**

The bold-type marks statistically significant values of linear correlation coefficient at the confidence level of 95%

In areas built of crystalline rocks, such as the authors’ study area, waters supplied to households and used for consumption are drawn from springs or shallow wells. Such intakes are usually situated in dislocation zones transporting groundwater from fractured rocks representing local or transitional flow system. The only alternative is collecting surface waters, which are usually definitely inferior in quality. In compliance with European and Polish law (Council Directive, 2013; Ordinance of the Minister of Health, 2017), radon waters flowing from three (K1, K3 and K4) out of the six studied springs cannot be supplied to households or consumed without prior radon removal. As stated by Polish law, waters from the other three springs should be also monitored for ^222^Rn activity concentration (Ordinance of the Minister of Health, 2017).

At the same time, radon groundwaters flowing out in springs K1, K3 and K4, as well as low-radon waters from spring CzS can be regarded as potentially medicinal. However, low-radon waters flowing out in CzS spring may periodically not satisfy the requirements for being recognized as medicinal radon waters, as ^222^Rn activity concentration in them drops below 74 Bq/L. Nevertheless, according to Polish law (Act, 2011), groundwaters flowing out in springs K1, K3 and K4 may be recognized as medicinal radon waters based on the results of more detailed investigations. They could then be used in radon therapy or radon balneotherapy treatments (Przylibski, 2018). Summing up, the obtained results indicate that the research area can be regarded as an area of potentially medicinal radon water occurrence. This is the second area of this kind identified in the Orlica-Bystrzyca metamorphic complex (Fig. 3). It seems likely that further research in the area of this geological unit will enable proving the existence of other areas with potentially medicinal radon waters. It is also possible that all the Orlica-Bystrzyca metamorphic complex will be able to be regarded as an area with occurrence of potentially medicinal radon waters. At the same time, such areas, or the whole Orlica-Bystrzyca metamorphic complex, being an area of medium radon potential, should be recognized as an area where groundwaters must be subject to detailed analysis of ^222^Rn content dissolved in them before being used in households and for consumption. If necessary, ^222^Rn activity concentration in these waters should be monitored or they should be purified, i.e. de-radoned, so that ^222^Rn content in them will be lower than 100 Bq/L before they are supplied to a pipe network.

## Conclusions

In the studied area of the Orlica-Bystrzyca metamorphic complex situated in the Bystrzyckie Mountain range, which is entirely an area of medium radon potential, occurrence of low-radon waters has been identified in three springs, and of radon waters—in another three. ^222^Rn activity concentration in groundwaters discharged from these springs oscillated between 35.3 and 272.0 Bq/L. The ^222^Rn originates entirely from dissolution in the studied groundwaters of gas formed in their reservoir rocks as a result of parent ^226^Ra decay. Taking account of the obtained results and archival data, a new value of the hydrogeochemical background of ^222^Rn in groundwaters in the Orlica-Bystrzyca metamorphic complex was calculated. It is 10–317 Bq/L. The basic statistical parameters of the concentration of ^222^Rn dissolved in groundwaters in the Orlica-Bystrzyca metamorphic complex, i.e. the minimum, the maximum, the mean and the median are 0.3, 448.0, 107.6 and 67.0 Bq/L, respectively.

The taken measurements have confirmed the mosaic-like image of spatial distribution of ^222^Rn concentration in groundwaters occurring in areas built of crystalline rocks. This fact had already been described in earlier publications from the Sudetes, as well as from certain regions of India and Canada. The authors have also found out that it is probably the presence of brittle tectonic deformations in the vicinity of springs that has a decisive influence on ^222^Rn activity concentration in the studied groundwaters, while the role of lithology is only secondary.

The authors have also observed a statistically significant strong correlation between ^222^Rn activity concentration and spring discharge Q in low-radon water and radon water in springs CzS and K4, respectively. Along with an increase in spring discharge, ^222^Rn activity concentration increases too, which means that radon content rises with an increase in water flow intensity in the water-bearing zone near the intake. A rise in water flow velocity results in the ability of ^222^Rn released from reservoir rocks and dissolved in the flowing groundwater to travel with this water over a longer distance within the time before its decay, which is constant and equals about 38.2 days. This means that dissolved ^222^Rn may reach a spring from a longer distance; hence, its activity concentration is higher, leading to an increase in the capacity of the recharge zone supplying the spring with ^222^Rn. This observation is consistent with previous findings from the Sudetes, including those from the areas of Świeradów-Zdrój Spa and Lądek-Zdrój Spa. However, this regularity has not been confirmed by observations of K6 spring in the studied area, in which no correlation between ^222^Rn activity concentration and discharge Q has been found. The area recharging this spring in groundwater and ^222^Rn requires further investigations. At the same time, a statistically significant inversely proportional correlation was found between ^222^Rn activity concentration and water temperature T in all the springs (CzS, K4 and K6). This means that the lower the temperature of water, the higher the activity concentration of ^222^Rn dissolved in it. The causes of such a relationship should be sought in the groundwater flow system. It is likely that precipitation waters cause the expansion of reservoir rock washing zone and an increase in the velocity of water flowing to the spring, which results in an increase in ^222^Rn activity concentration in water appearing in the spring. Assuming the piston flow model, it can be expected that fresh rainwater displaces the significantly saturated with ^222^Rn older water from the source reservoir. However, in order to confirm such a mechanism, a much detailed measurements would be necessary.

In areas with medium radon potential, where ^222^Rn activity concentration in soil air ranges from 10 to 50 kBq/m^3^, radon groundwaters may be common. This is the reason why all groundwaters which may be intended for human consumption or household use in such areas should be subject to obligatory monitoring of ^222^Rn activity concentration. In the event of recognizing occurrence of radon waters, i.e. waters with ^222^Rn activity concentration of at least 100 Bq/L, purification of such water by removing radon is necessary before supplying it to the pipe network. The simplest methods of de-radoning are aeration of the water, which also allows the removal of iron, or the use of filters with an activated carbon layer, which also remove other undesirable components. Both of these methods are used routinely to treat water intended for human consumption. If ^222^Rn activity concentration of at least 1000 Bq/L is recorded (high-radon and extreme-radon waters), such groundwater should not be intended for household use or human consumption.

At the same time, the research area can be regarded as an area of potentially medicinal radon water occurrence. It is the second area of this kind identified in the Orlica-Bystrzyca metamorphic complex, one of six in the Kłodzko basin, and the twelfth in the Polish part of the Sudetes. It seems likely that further research in the area of this geological unit will enable proving the existence of other areas with potentially medicinal radon waters. It is also possible that the entire Orlica-Bystrzyca metamorphic complex will be able to be regarded as an area with potentially medicinal radon water occurrence. Such an observation justifies a claim that in areas with medium radon potential, groundwaters which are not suitable as a source of water for the population because of too high ^222^Rn activity concentration in them can be used as medicinal radon waters in therapeutic treatments. Also, radon obtained from these waters may be used in other radon therapy treatments. With this aim, detailed research into radon waters should be conducted, and the usage or these waters or radon obtained from them should be strictly controlled by qualified balneologists.

The results obtained by the authors indicate a likelihood of wide spatial variation in the activity concentration of ^222^Rn dissolved in groundwaters in areas built of various crystalline (metamorphic and igneous) rocks. In areas of this type (crystalline massifs on all continents), one should expect the occurrence of radon-enriched groundwaters. It is necessary then to remember about de-radoning them before they are used for human consumption. At the same time, it should be remembered that radon-enriched groundwaters can be used as medicinal waters in many countries. Radon waters then become a valuable resource, which can restore people’s health. Therefore, areas built of crystalline rocks, often having high or medium radon potential, can be a source of radon waters not infrequently used in balneotherapy treatments in health resorts.

## Data Availability

The authors confirm that the data supporting the findings of this study are available within the article and in references and are available from the corresponding author, [TAP], upon reasonable request.

## References

[CR1] Act of June 9, 2011, Geological and mining law. Consolidated text, Dz. U. z dnia 9 lutego 2015 r., poz. 196. (in Polish).

[CR2] Adamczyk-Lorenc A. (2007) Hydrogeochemical background of radon in groundwaters of the Sudetes, Ph.D. Thesis, Wrocław University of Technology, Faculty of Geoengineering, Mining and Geology, Wrocław (unpublished; in Polish).

[CR3] Becker K (2004). One century of radon therapy. International Journal of Low Radiation.

[CR4] Bellotti E, Broggini C, DiCarlo G, Laubenstein M, Menegazzo R (2015). Precise measurement of the ^222^Rn half-life: A probe to monitor the stability of radioactivity. Physics Letters B.

[CR5] Buczyński S, Staśko S (2016). Groundwater flow systems in the sudeten mountains: A study of the Kamieńczyk catchment area. Episodes.

[CR6] Chau ND, Duliński M, Jodłowski P, Nowak J, Różański K, Sleziak M, Wachniew P (2011). Natural radioactivity in groundwater – a review. Isotopes in Environmental and Health Studies.

[CR7] Cho BW, Choo ChO (2019). Geochemical behavior of uranium and radon in groundwater of Jurassic granite area, Icheon Middle Korea. Water.

[CR8] Ciężkowski W, Przylibski TA (1997). Radon in waters from health resorts of the Sudety Mts. (SW Poland). Applied Radiation and Isotopes.

[CR9] Council Directive 2013/51/EURATOM of 22 October 2013 laying down requirements for the protection of the health of the general public with regard to radioactive substances in water intended for human consumption. Official Journal of the European Union, 7.11.2013., L 296/12 – L 296/21.

[CR10] Cucu A, Shreder K, Kraft D, Rühle PF, Klein G, Thiel G, Frey B, Gaipl US, Fournier C (2017). Decrease of markers related to bone erosion in serum of patients with musculoskeletal disorders after serial low-dose radon spa therapy. Frontiers in Immunology.

[CR11] Danzer R., Lube T. (1996) New Fracture Statistics for Brittle Materials. [in:] R.C. Bradt, D.P.H. Hasselmann. D. Munz, M. Sakai, V. Yashevchenkov [ed.]: Fracture mechanics of ceramics, Vol. 11, Plenum Publishing Corp., New York, pp. 425–439.

[CR12] Don J, Dumicz M, Wojciechowska I, Zelazniewicz A (1990). Lithology and tectonics of the Orlica-Snieznik Dome, Sudetes-Recent state of knowledge. Neues Jahrbuch für Geologie und Paläontologie. Abhandlungen.

[CR13] Erickson BE (2006). Range of motion assessment of elderly arthritis sufferers at Montana (USA) Radon Health Mines. International Journal of Low Radiation.

[CR14] Erickson BE (2007). The therapeutic use of radon: A biomedical treatment in Europe; An „alternative” remedy in the United States. Dose-Response.

[CR15] Falkenbach A, Kovacs J, Franke A, Jörgens K, Ammer K (2005). Radon therapy for the treatment of rheumatic diseases – review and meta-analysis of controlled clinical trials. Rheumatology International.

[CR16] Franke A, Reiner L, Pratzel HG, Franke T, Resch KL (2000). Long-term efficacy of radon spa therapy in rheumatoid arthritis – a randomised, sham-controlled study and follow-up. Rheumatology.

[CR17] Franke A, Reiner L, Resch KL (2007). Long-term benefit of radon spa therapy in the rehabilitation of rheumatoid arthritis: A randomised, double-blinded trial. Rheumatology International.

[CR18] Freiler Á, Horváth Á, Török K, Földes T (2016). Origin of radon concentration of Csalóka Spring in the Sopron Mountains (West Hungary). Journal of Environmental Radioactivity.

[CR19] Girault F., Perrier F., Przylibski T.A. (2018) Radon-222 and radium-226 occurrence in water: a review. [in:] Gillmore, G.K., Perrier, F.E. & Crockett, R.G.M. (eds): Radon, Health and natural hazards. Geological Society, London, Special Publications, 451, pp. 131–154. 10.1144/SP451.3.

[CR20] Janica D. (2002), Natural hydrogeochemical background of the Quaternary groundwaters of north-eastern Poland. Ph.D. Thesis, University of Warsaw, Faculty of Geology, Warszawa (in Polish).

[CR21] Kapetanović A, Hodžić S, Avdić D (2013). The effect of mineral radon water applied in the form of full baths on blood pressure in patients with hypertension. Journal of Health Sciences.

[CR22] Krasny J (1993). Classification of transmissivity magnitude and variation. Ground Water.

[CR23] Lachassagne P, Wyns R, Berard P, Bruel T, Chery L, Coutand T, Desprats JF, Le Strat P (2001). Exploitation of high-yields in hard rock aquifers: Downscaling methodology combining GIS and multicriteria analysis to delineate field prospecting zones. Ground Water.

[CR24] Martins LMO, Pereira AJSC, Sousa OA, Sanches Fernandes LF, Pacheco FAL (2020). A new radon prediction approach for an assessment of radiological potential in drinking water. Science of the Total Environment.

[CR25] Mazur S, Aleksandrowski P, Kryza R, Oberc-Dziedzic T (2006). The Variscan orogen in Poland. Geological Quarterly.

[CR26] Mazur S, Aleksandrowski P, Szczepański J (2010). Outline structure and tectonic evolution of the Variscan Sudetes. Przegląd Geologiczny.

[CR27] Modelska M, Buczyński S, Staśko S (2015). Chemical composition of groundwater of Kamieńczyk catchment area in Sudety Mts. Przegląd Geologiczny.

[CR28] Moder A, Hufnagl C, Jakab M, Hitzl W, Ritter M (2011). Radon-therapy in ankylosing spondylitis reduces auto-antibody titers. Open Journal of Molecular and Integrative Physiology.

[CR29] Nagy K, Kávási N, Kovács T, Somlai J (2008). Radon therapy and speleotherapy in Hungary. La Presse Thermale Et Climatique.

[CR30] Ordinance of the Minister of Health of December 7, 2017 on the quality of water intended for human consumption. Dz. U. z dnia 11 lutego 2017 r., poz. 2294 (in Polish).

[CR31] Persianova-Dubrova AL, Badalov NG, Lvova NV, Tupitsyna IU, Uianaeva AI, Krikorova SA, Adilov VB, Linok VA, Povazhnaia EL (2012). Crenobalneotherapy in Russia. La Presse Thermale Et Climatique.

[CR32] Piao Ch, Tian M, Gao H, Gao Y, Ruan J, Wu L, Gao G, Yi L, Liu J (2020). Effects of radon from hot springs on lymphocyte subsets in peripheral blood. Dose-Response.

[CR33] Pinti DL, Retailleau S, Barnetche D, Moreira F, Moritz AM, Larocque M, Gélinas Y, Lefebvre R, Hélie J-F, Valadez A (2014). ^222^Rn activity in groundwater of the St. Lawrence Lowlands, Quebec, eastern Canada: relation with local geology and health hazard. Journal of Environmental Radioactivity.

[CR34] Przylibski TA (2000). Estimating the radon emanation coefficient from crystalline rocks into groundwater. Applied Radiation and Isotopes.

[CR35] Przylibski TA (2000). Size estimation and protection of the areas supplying radon to groundwater intakes. Archives of Environmental Protection.

[CR36] Przylibski TA (2000). ^222^Rn concentration changes in medicinal groundwaters of Lądek Zdrój (Sudety Mountains, SW Poland). Journal of Environmental Radioactivity.

[CR37] Przylibski T.A. (2005) Radon. Specific component of medicinal waters in the Sudety Mountains. Oficyna Wydawnicza Politechniki Wrocławskiej, Wrocław (in Polish).

[CR38] Przylibski TA (2011). Shallow circulation groundwater – the main type of water containing hazardous radon concentration. Natural Hazards and Earth System Sciences.

[CR39] Przylibski TA (2015). Radon research in Poland: A review. Solid State Phenomena.

[CR40] Przylibski T.A. (2018) Radon. A radioactive therapeutic element. [in:] Gillmore, G.K., Perrier, F.E. & Crockett, R.G.M. (eds): Radon, health and natural hazards. Geological Society, London, Special Publications, 451, pp. 209–236. 10.1144/SP451.7.

[CR41] Przylibski TA, Żebrowski A (1999). Origin of radon in medicinal waters of Lądek Zdrój (Sudety Mountains, SW Poland). Journal of Environmental Radioactivity.

[CR42] Przylibski TA, Kozłowska B, Dorda J, Kiełczawa B (2002). Radon-222 and ^226^Ra concentrations in mineralized groundwaters of Gorzanów (Kłodzko Basin, Sudeten Mountains, SW Poland). Journal of Radioanalytical and Nuclear Chemistry.

[CR43] Przylibski TA, Staśko S, Szczepanowski S, Modelska M, Dorda J, Kozłowska B (2002). Preliminary results of determinations of radon and radium concentrations in surface and underground waters in the upper part of the Kamienica River catchment basin (Śnieżnik Massif, Sudetes, SW Poland). Przegląd Geologiczny.

[CR44] Przylibski T.A. (ed.), Adamczyk-Lorenc A., Żak S. (2007) Areas of the occurrence of potentially medicinal radon waters in Sudetes. Part II [in]: Wołkowicz S. (ed.): Radon potential of the Sudetes with determination of potentially medicinal radon water areas. Państwowy Instytut Geologiczny, Warszawa. (in Polish with English summary).

[CR45] Przylibski TA, Fijałkowska L, Bielecka A (2008). Potentially medicinal radon waters of Ślęża Massif. Przegląd Geologiczny.

[CR46] Przylibski TA, Gorecka J, Kula A, Fijałkowska-Lichwa L, Zagożdżon K, Zagożdżon P, Miśta W, Nowakowski R (2014). ^222^Rn and ^226^Ra activity concentrations in groundwaters of southern Poland: New data and selected genetic relations. Journal of Radioanalytical and Nuclear Chemistry.

[CR47] Sawicki L. (1962) Detailed geological map of the Sudetes, sheet Międzylesie. Scale 1 : 25 000, Wydawnictwa Geologiczne, Warszawa. (in Polish).

[CR48] Sawicki L. (1995) Geological map of Lower Silesia region with adjacent areas of Czech Republic and Germany (without Quaternary deposits). Scale 1 : 100 000. Warszawa: Państwowy Instytut Geologiczny.

[CR49] Seminsky KZh, Seminsky AK (2016). Radon in groundwaters in the Baikal region and Transbaikalia: Variations in space and time. Geodynamics & Tectonophysics.

[CR50] Somlai J, Kávási N, Szabó T, Várhegyi A, Kovács T (2007). The function of radon in curing respiratory diseases in the therapeutic cave of Tapolca. Journal of Radioanalytical and Nuclear Chemistry.

[CR51] Staśko S (2002). Water-bearing capacity of hard rocks in the Sudetes. Biuletyn PIG.

[CR52] Staśko S., Tarka R. (2002) Groundwater recharge and drainage processes in mountainous terrains based on research in the Śnieżnik Massif (Sudetes, SW Poland). Acta Univ. Wratisl., Prace Geol.-Mineral., 2528: 1–86. (in Polish with abstract in English).

[CR53] Sukanya S, Noble J, Joseph S (2021). – Factors controlling the distribution of radon (^222^Rn) in groundwater of a tropical mountainous river basin in southwest India. Chemosphere.

[CR54] Szczepańska J., Kmiecik E. (1998) Statistical control of data quality in groundwater monitoring. Wydawnictwa AGH, Kraków. (in Polish).

[CR55] Szczepański J. (2010) Provenance and tectonometamorphic evolution of the supracrustal series from the Bystrzyckie Mts. crystalline massif. Uniwersytet Wrocławski, WDN PAN, Wrocław.

[CR56] Thivya C, Chidambaram S, Thilagavathi R, Tirumalesh K, Nepolian M, Prasanna MV (2017). Spatial and temporal variations of radon concentrations in groundwater of hard rock aquifers in Madurai district, India. Journal of Radioanalytical and Nuclear Chemistry.

[CR57] Vogiannis E, Nikolopoulos D, Louizi A, Halvadakis CP (2004). Radon variations during treatment in thermal spas of Lesvos Island (Greece). Journal of Environmental Radioactivity.

[CR58] Voronov AN (2004). Radon-rich waters in Russia. Environmental Geology.

[CR59] Walencik-Łata A, Kozłowska B, Dorda J, Przylibski TA (2016). The detailed analysis of natural radionuclides dissolved in spa waters of the Kłodzko Valley, Sudety Mountains, Poland. Science of the Total Environment.

[CR60] Walia V, Bajwa BS, Virk HS (2003). Radon monitoring in groundwater of some areas of Himachal Pradesh and Punjab states, India. Journal of Environmental Monitoring.

[CR61] Wołkowicz S. (ed.) (2007) Radon potential of the Sudetes with determination of potentially medicinal radon water areas. Państwowy Instytut Geologiczny, Warszawa. (in Polish with English summary).

[CR62] Zdrojewicz Z, Strzelczyk J (2006). Radon treatment controversy. Dose-Response.

[CR63] Żelaźniewicz A. (2015) Geological past in the nature of Lower Silesia, PAN Wrocław. (in Polish).

